# 1830. Epidemiology of Recurrent Bacterial Bloodstream Infections in the US Military Health System

**DOI:** 10.1093/ofid/ofac492.1460

**Published:** 2022-12-15

**Authors:** Alexander C Vostal, Melissa Grance, John H Powers, Leigh Carson, Uzo Chukwuma, Charlotte Lanteri, Nicholas Seliga, Beth Poitras, Edward Parmelee, Katrin Mende

**Affiliations:** National Institute of Allergy and Infectious Diseases, Silver Spring, Maryland; Infectious Disease Clinical Research Program, Department of Preventive Medicine and Biostatistics, Uniformed Services University of the Health Sciences, Bethesda, Maryland; Leidos Biomedical Research, Rockville, Maryland; Infectious Disease Clinical Research Program, Bethesda, Maryland; Navy and Marine Corps Public Health Center, Rockville, Maryland; Infectious Disease Clinical Research Program, Department of Preventive Medicine and Biostatistics, Uniformed Services University of the Health Sciences, Bethesda, Maryland; Navy and Marine Corps Public Health Center, Rockville, Maryland; Navy and Marine Corps Public Health Center, Rockville, Maryland; Infectious Disease Clinical Research Program, Department of Preventive Medicine and Biostatistics, Uniformed Services University of the Health Sciences, Bethesda, Maryland; Infectious Disease Clinical Research Program, Department of Preventive Medicine and Biostatistics, Uniformed Services University of the Health Sciences, Bethesda, MD, USA, San Antonio, Texas

## Abstract

**Background:**

The epidemiology of recurrent bacterial bloodstream infections (rBSI) has not been fully characterized. Evaluating rBSI represents opportunities to inform morbidity risk factors and prevention strategies. We describe the clinical and microbiological features of rBSI in the US Military Health System (MHS) in a prospective cohort study, including retired and active-duty US uniformed service members and their beneficiaries.

**Figure:**
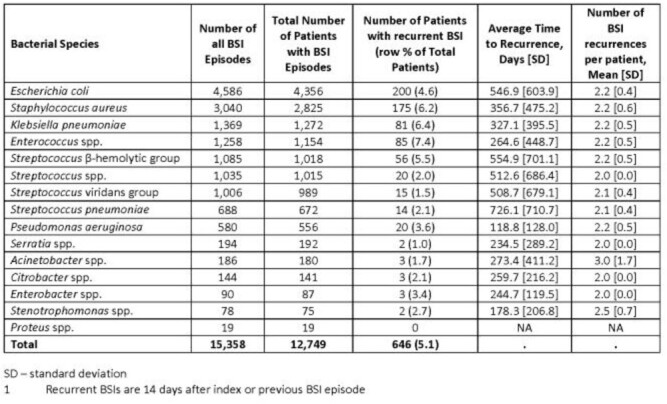

**Methods:**

We collected data for rBSI episodes from MHS beneficiaries (Jan 2010 – Dec 2019). A rBSI is defined as growth of the same bacterial pathogen in blood culture >14 days after the index or previous episode. Demographics and comorbidities were collected prior to the index BSI. Microbiological data were obtained from the Navy and Marine Corps Public Health Center. Descriptive statistics are presented.

**Results:**

A total of 12,749 beneficiaries were diagnosed with a BSI attributed to 1 of the 15 most common bacterial pathogens associated with BSI in the MHS, with 646 (5.1%) experiencing a rBSI. *Escherichia coli* had the largest proportion among all patients with rBSI (31% of 646); however, *Enterococcus* spp. accounted for the highest proportion of rBSI within a given pathogen subgroup (7.4% of 1,154 *Enterococcus* BSI; Table). *Pseudomonas aeruginosa* BSI had the shortest average time to recurrence (119 days), and *Acinetobacter* spp. had the highest frequency of BSI recurrences per patient (mean of 3). Male sex (59.9%) and age ≥65 years (52.9%) were most common among the rBSI patients. The updated Charlson Comorbidity index score preceding the index BSI was a median of 5.0, and chronic pulmonary disease (57.3%) and diabetes (56.6%) contributed the largest proportion of common comorbidities. A total of 88 (13%) rBSI patients had their index BSI while hospitalized following trauma where *S. aureus* was the most common (37.5%) bacterial pathogen.

**Conclusion:**

Overall, the proportion of rBSI (5.1%) in our cohort of MHS beneficiaries was generally lower than that previously reported in the literature. Individuals with rBSI had a substantial burden of comorbid disease with 13% having trauma precede the index BSI. Identifying risk factors for recurrence may improve management strategies of primary BSI and may reduce morbidity of subsequent BSI.

**Disclosures:**

**John H. Powers, III, MD**, Arrevus: Advisor/Consultant|Eicos: Advisor/Consultant|Evofem: Advisor/Consultant|Eyecheck: Advisor/Consultant|Gilead: Advisor/Consultant|GlaxoSmithKline: Advisor/Consultant|OPKO: Advisor/Consultant|Resolve: Advisor/Consultant|Romark: Advisor/Consultant|SpineBioPharma: Advisor/Consultant|UTIlity: Advisor/Consultant|Vir: Advisor/Consultant.

